# Efficiency of Trichome-Based Plant Defense in *Phaseolus vulgaris* Depends on Insect Behavior, Plant Ontogeny, and Structure

**DOI:** 10.3389/fpls.2017.02006

**Published:** 2017-11-24

**Authors:** Zhenlong Xing, Yongqiang Liu, Wanzhi Cai, Xinzheng Huang, Shengyong Wu, Zhongren Lei

**Affiliations:** ^1^State Key Laboratory for Biology of Plant Diseases and Insect Pests, Institute of Plant Protection, Chinese Academy of Agricultural Sciences, Beijing, China; ^2^Department of Entomology, China Agricultural University, Beijing, China; ^3^Fujian-Taiwan Joint Center for Ecological Control of Crop Pests, Fujian Agriculture and Forestry University, Fuzhou, China

**Keywords:** trichome-based defense, trapping behavior, *Liriomyza trifolii*, capture efficiency, observation on trapped body parts

## Abstract

Plant trichomes often function as physical barriers in preventing arthropod feeding and oviposition. Even though insects are frequently reported being entrapped and killed by trichome traps, the actual trapping behavior has not yet been described in detail. Capture experiments showed that capture efficiency during the plant's vegetative stage was considerably higher than in the fruiting and cotyledon stages. The ventral surface of the leaf was more effective in trapping flies than other parts of the plant. Capture-events monitoring showed that the mouthparts, legs, and ovipositor of *Liriomyza trifolii* adults are the body parts involved in entrapment by surface trichomes on *Phaseolus vulgaris* plants, and subsequently, deter their ability to feed, walk, and oviposit. Of the three main body parts normally affected, mouthparts was found to be the body part most susceptible to the trichomes. Entrapments were most often caused by landing, followed by puncturing or feeding, and occasionally by walking or fighting. Using scanning electron microscopy (SEM) and optical microscopy, we determined the susceptible positions of each body part and found that the flies were all trapped by hooked trichomes. This study revealed the process by which leafminer flies are entrapped by surface trichomes of the host plant and evaluated the capture efficiency. The results will contribute to our understanding of physical defenses against herbivores.

## Introduction

Interactions between plants and insects include some of the most interesting and important interactions between species. Plant defenses against herbivores, which are often remarkably complex and diverse, can be mechanical or chemical, direct, or indirect (War et al., 2012). The surfaces of terrestrial plants have evolved many morphological adaptations (including a waxy cuticle and/or spines, setae, or trichomes that developed from epidemic cells) to cope with biotic challenges, and comprise the first line of plant defenses (Lazniewska et al., 2012; Hauser, 2014).

Trichomes, which have developed on nearly all non-aquatic plant structures such as leaves, stems and even fruits, can be glandular or non-glandular, unicellular or multicellular, and often serve as physical barriers against insect attack and fungal infection (Levin, 1973; Lazniewska et al., 2012). The glandular trichomes are able to secrete adhesive or viscous fluids that act to entrap arthropods or discourage herbivore feeding (Wagner, 1991; Wheeler and Krimmel, 2015). In addition, the entrapped victims of the sticky plants may attract predatory enemies of the herbivores to enhance the plant's indirect defenses (Krimmel and Pearse, 2013). Non-glandular trichomes include types consisting of a spine or are hooked at various angles that are capable of directly impaling insect bodies and thereby impeding the insects' feeding behavior (Levin, 1973; Riddick and Simmons, 2014).

Non-glandular hooked trichomes are considered to be specific structures that are effective in trapping a multitude of herbivores as well as their natural enemies (Eisner et al., 1998; Riddick and Simmons, 2014). The entrapments do have a direct impact on individual fitness and population evolution of the affected insects (Levin, 1973; Riddick and Simmons, 2014; Peterson et al., 2016). For example, hooked trichomes can reduce an aphids' longevity and reproduction rate and increase their nymphal mortality (Johnson, 1953). There are also instances of anti-defensive traits evolving in some specialist herbivores, such as a butterfly larva being able to spin a fine silk covering over trichomes to facilitate its passage (Rathcke and Poole, 1975). Furthermore, the presence of trichomes may influence host plant selection by herbivores, where plants with lower trichome densities could be selectively exploited by herbivores (Pang et al., 2004, 2006).

The entrapment is the result of interactions between insect behaviors and the physical structures and chemical defenses of plant trichome traps. Captures by hooked trichomes were always assumed to occur when insects visited (i.e., crawling or walking across) the trichome traps (Gilbert, 1971; Levin, 1973; Eisner et al., 1998; Cardoso, 2008), although the specific trapping behavior involved in trichome-based plant defensive systems, that is, how the insects are actually entrapped by the hooklike hairs, has never been fully described.

The vegetable leafminer, *Liriomyza trifolii* (Burgess) (Diptera: Agromyzidae), which was introduced into China in 2005 and has since gradually expanding throughout the southeastern regions of the country, is a highly invasive and polyphagous pest of many vegetable and ornamental plant species that is especially attracted to various bean plant cultivars (Lei et al., 2007; Yang et al., 2010). The non-glandular trichomes found on kidney beans (*Phaseolus vulgaris* L.), which can be straight or hooked with variable densities and distributions on the plant, are variety-dependent (Stenglein et al., 2004; Jimenez et al., 2012). The trichomes function as typical physical barriers against many agricultural pests, such as leafhoppers (Pillemer and Tingey, 1976) and aphids (Johnson, 1953). Although a previous study by Quiring et al. (1992) reported that hooked trichomes found in bean plants were able to reduce the average lifespan of adult *L. trifolii* females, and the trichome density was correlated with different parts of the bean leaflet, the precise sequence of fly behaviors leading to entrapment by surface trichomes remains unclear.

This study was designed to elucidate the precise trapping behavior involved in this capture phenomenon. Our study involved evaluating the trapping efficiency during three stages of plant development, followed by detailed monitoring and analysis of the trapping behavior using a continuous monitoring system. Finally, we examined the physical trichome insertions and/or hooking process on each body region using SEM and optical microscopy.

## Materials and methods

### Insect culture

Populations of *L. trifolii* were initially collected from cowpea fields in Sanya, Hainan Province, China in 2015, and subsequently cultured on kidney beans, *P. vulgaris* L., at 26 ± 2°C, RH: 75% with a 14:10 light: dark photoperiod. The populations were reared for more than three generations to ensure homogeneity of the *L. trifolii* cultures prior to their use in the experiments. Freshly emerged adults were used in each of the following studies.

### The plant

The kidney beans (*P. vulgaris*) were grown in plastic containers inside a glasshouse, with two plants per pot (12 cm diam., 10 cm high). Three distinct stages of the plant were utilized in this study: the cotyledon stage (after the primary leaves had fully expanded), the leaf stage (after the compound leaves had fully expanded), and the fruiting stage (after the bean pods had developed to ~10 cm in length).

### Trapping experiments

One container of healthy and uniform bean plants was selected and placed inside a nylon mesh cage (30 ^*^ 30 ^*^ 40 cm) to initiate the experiments. The environmental conditions were maintained at 26 ± 2°C, RH: 50% with a 14:10 light: dark photoperiod.

We selected 20 early emerging and healthy adults for each replicate of the three plant growth stages. There were 14, 9, and 16 replicates for the cotyledon, leaf, and fruiting stages, respectively.

After releasing the flies in each replicate, we checked every 2 h during daylight hours (*L. trifolii* activity is diurnal) (Chandler, 1985) and counted the number of leafminers that had died as a consequence of trichome entrapment (the position of every observed captured fly was marked with a marker pen to ensure the counted dead flies had actually died as a consequence of entrapment). Each of the observations was concluded after all of flies had died. The percentage of entrapped flies relative to the total number of released flies was calculated as the capture mortality (Pillemer and Tingey, 1976).

### Trapping behavior monitoring in real time

The trapping behavior of insects was observed using a real-time network video recorder (HIKVISION DS7832, Hangzhou, China) attached to a video camera (HIKVISION DS2CD4032FWD, Hangzhou, China) (Lei et al., 2016). We selected the most susceptible areas of the plants during the leaf stage—the ventral surface of the fully expanded middle leaflet of healthy and uniform plants, to monitor trapping behavior.

A clear plastic cup (10 cm diam., 16 cm high) covered with a transparent plexiglass shutter (12 ^*^ 12 cm) was used to observe the trapping behavior. After the petiole was inserted into a vial of water outside of the cup to maintain moisture, the camera was focused on the ventral surface of the leaf (Lei et al., 2016). After arranging the setup, 20 early emerging and healthy flies were collected, anesthetized with CO_2_, and carefully placed into the observation cup. Monitoring was conducted at 26 ± 2°C, with a 24 h light (21 watt) to enable video recording.

Each replicate was started between 14:00 and 16:00 p.m. because the majority of leafminer flies would emerge prior to 14:00 p. m. The video monitorings were continued for 48 h after each release, and replicated 15 times.

After the monitoring ended, the video was rerun, frame-by-frame, in an attempt to record every capture event. In each capture, four parameters were recorded: (1) the initial time of flies being trapped; (2) the trapping behavior causing the fly to be trapped; (3) which body part(s) was (were) trapped; and, (4) whether the fly was able to successfully escape or not, and if so, the time of escape was recorded. The percentage of escaped flies in the total number of entrapped flies was calculated as escape rate.

### Observation of captured fly body parts

#### SEM observation

All SEM imaging was performed using an FEI Quanta 200 FEG ESEM (FEI, Hillsboro, Oregon, USA) under low-vacuum mode (LV-SEM). Entrapped flies on leaves and stems were prepared by cutting the leaf around the entrapped leafminer to a size approximating the size of an SEM stub, and mounting the leaf piece with its attached leafminer on the SEM stub with copper tape (Szyndler et al., 2013). Due to the difficult angle involved in viewing trapped mouthparts and ovipositors, SEM observations were only suitable for viewing trapped legs.

#### Optical microscopy observations

Observations of trapped mouthparts and ovipositors were performed with an optical microscope (Olympus BX51, Olympus America Inc., http://www.olympusmicro.com) equipped with a DP72 imaging system. All observations were done under transmitted light. Entrapped flies on leaves were prepared by cutting the leaf around the entrapped leafminer to a size suitable for a microslide, and mounting the leaf piece with its attached leafminer on a microslide with double sided adhesive tape. Since the trapped leafminer flies were still alive and continued to struggle, we occasionally needed to adjust the flies with a micro-forcep to facilitate our observations when the trichomes were entrapped on the mouthparts or ovipositor. Video recordings were made after the trapping trichome(s) were located.

### Statistical analysis

Statistical analyses were conducted using IBM SPSS Statistics 19.0 (SPSS Inc., Chicago, IL, USA). All capture mortalities and escape frequencies were arcsine square root transformed before being analyzed (Warton and Hui, 2011). The effects of different plant growth stages and different plant regions on capture mortality were analyzed with two-way ANOVA followed by Tukey's HSD test at *P* < 0.05. Two indices were obtained from the capture monitoring videos: the number of capture events and escape rate. All capture monitoring data (i.e., the two indices of different capture behavior and different capturing body part) were independently analyzed using one-way ANOVA with Tukey's HSD test at *P* < 0.05. Graphs were drawn using Graphpad Prism 6 (Graphpad Software Inc., San Diego, California, USA) and Adobe Photoshop CS6 (Adobe Systems Incorporated, San Diego, California, USA).

### Ethical note

Our study involved populations of *L. trifolii*, which were maintained in a laboratory culture for over a year. The original collection locations were not privately owned or protected. No ethical approval or specific permissions were required for this study.

## Results

### Capture efficiency

**Figure 1:**
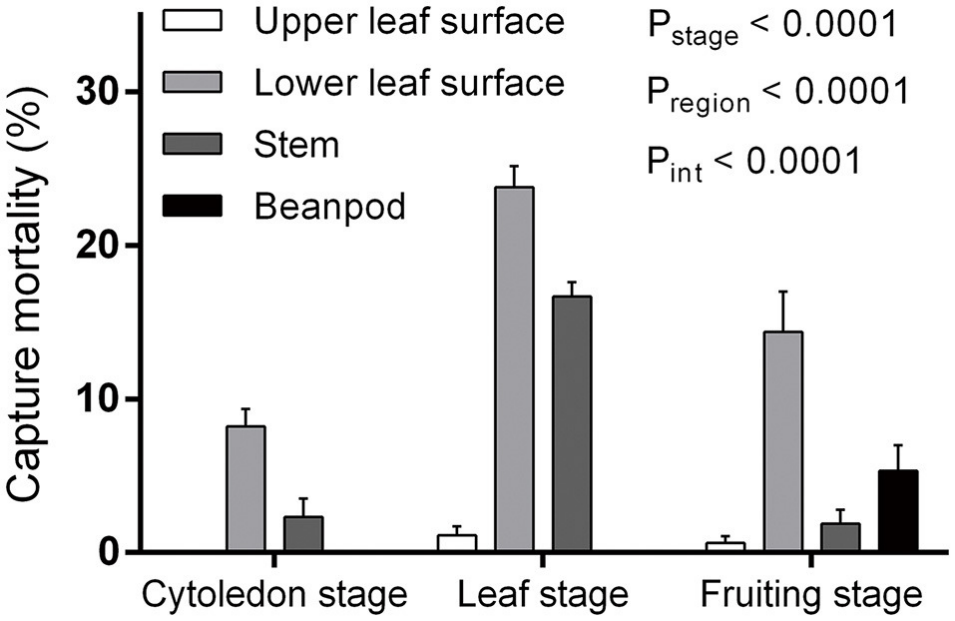
Capture mortality of *Liriomyza trifolii* adults during different developmental stages and on different regions of *Phaseolus vulgaris*. Error bars represent s.e.m.

Both plant growth stage and region had significant influence on capture efficiency, with a significant interaction between the two factors [*F*_(6, 123)_ = 6.46, *P* < 0.0001] (Figure 1). When comparing the three bean plant stages that were observed, capture mortality in the leaf stage (41.44 ± 2.39%, mean ± s.e.m., the same as follows) was higher than in the fruiting (21.88 ± 3.71%) and cotyledon (10.71 ± 1.56%) stages [*F*_(2, 123)_ = 24.62, *P* < 0.0001]. When different areas of the plant were compared, the lower leaf surface was the most efficient at trapping *L. trifolii* adults [*F*_(3, 123)_ = 47.93, *P* < 0.0001]. Compared to the lower leaf surface, the upper surface had the lowest capture efficiency, where no captured flies were found in the cotyledon stage. Interestingly, the bean pods in the fruiting stage were also able to successfully capture adult leafminer flies (Figure 1).

### Capture behaviors

We were able to define five distinct types of insect behaviors that resulted in entrapment: landing, puncturing + feeding, puncturing, walking/probing, and fighting (Video S1).

**Figure 2:**
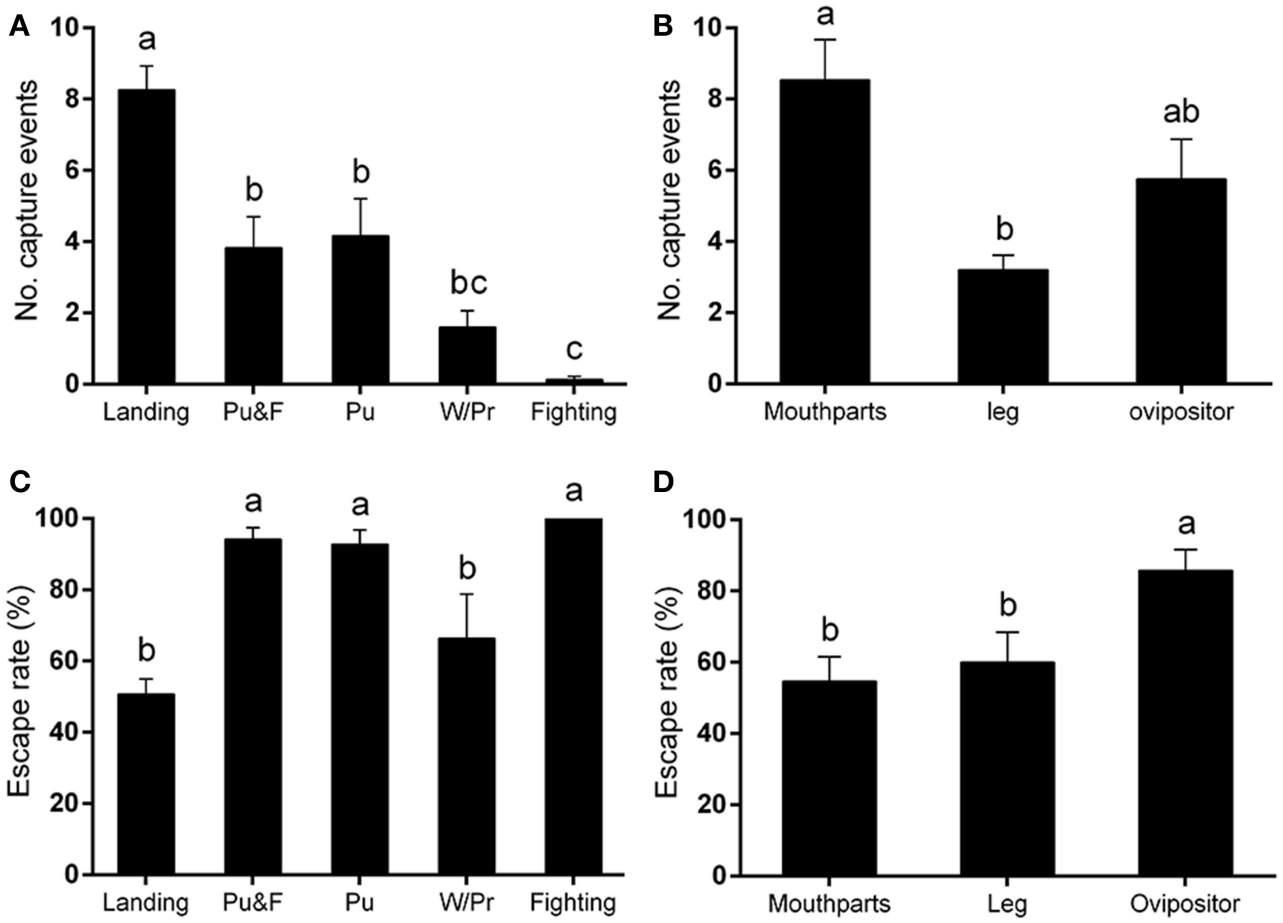
Capture behavior of *Liriomyza trifolii* adults on the ventral leaf surface of *Phaseolus vulgaris*. Capture events were continuously recorded for 48 h after release. **(A,B)** Number of capture events **(A)** caused by different fly behaviors, **(B)** affecting different fly body parts. **(C,D)** Escape rates after being trapped: **(C)** by different fly capture behaviors, **(D)** on different body parts. All values are mean ± s.e.m. Bars sharing the same letter mark indicate no significant differences were found at the 5% level using Tukey's HSD tests. “Pu” refers to flies captured during puncturing behavior; “Pu&F” refers to flies captured during puncturing and feeding behavior; “W/Pr” refers to flies captured during walking or probing behavior.

Among these five behaviors, landing was responsible for the most capture events, followed by puncturing, and puncturing + feeding behavior. Walking/probing while searching for suitable puncture sites for feeding also contributed to the entrapment totals. Other behaviors, including fighting for females or for food, would occasionally lead to the capture of leafminer flies according to video analysis [*F*_(4, 64)_ = 24.08, *P* < 0.0001] (Figure 2A).

In addition to the legs, the mouthparts and ovipositor of *L. trifolii* adults were frequently captured by the plant trichomes. In general, the mouthparts was the body part most susceptible to being entrapped (number of capture events: 8.53 ± 1.33). Leafminer flies were also commonly entrapped by their ovipositors (number of capture events: 5.75 ± 1.12), and, lastly, the legs were also susceptible to being entrapped (number of capture events: 3.20 ± 0.42) [*F*_(2, 39)_ = 8.81, *P* < 0.001] (Figure 2B).

**Figure 3:**
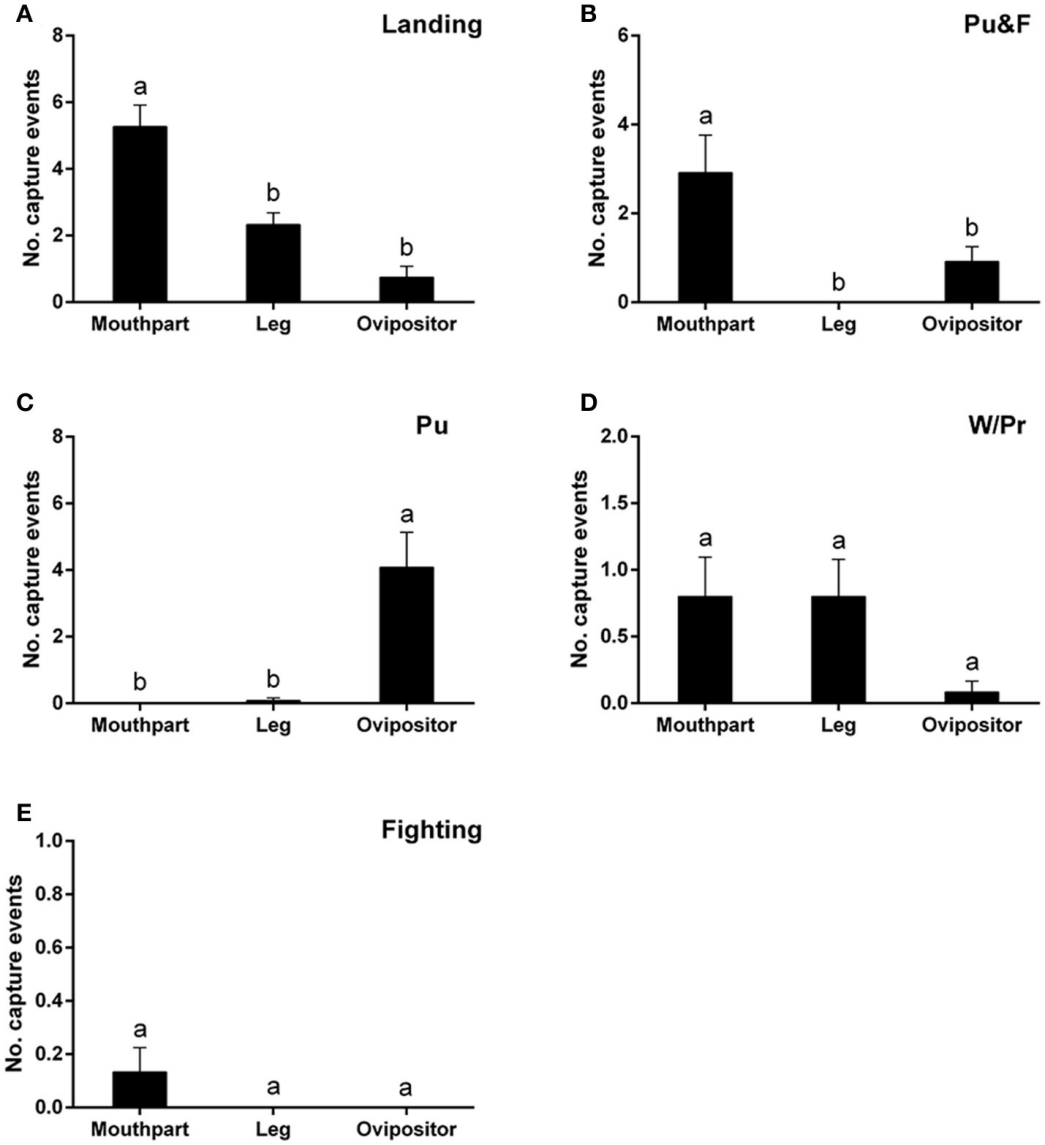
Number of capture events involving different body parts of *Liriomyza trifolii* adults on the ventral leaf surface of *Phaseolus vulgaris* caused by: Landing **(A)**, Pu&F **(B)**, Pu **(C)**, W/Pr **(D)**, and Fighting **(E)**. Capture events were continuously recorded for 48 h after release. All values are mean ± s.e.m. Bars sharing the same letter mark indicate no significant differences were found at the 5% level using Tukey's HSD tests. “Pu” refers to flies captured during puncturing behavior; “Pu&F” refers to flies captured during puncturing and feeding behavior; “W/Pr” refers to flies captured during walking or probing behavior.

Landing on the leaf surfaces contributed to most capture events and, in many instances, involved the mouthparts, although the legs and ovipositor were also be affected, to a lesser degree, by landing behavior [*F*_(2, 39)_ = 21.89; *P* < 0.0001] (Figure 3A). Puncturing behavior, accompanied by oviposition, would always lead to the ovipositor being trapped [*F*_(2, 33)_ = 14.79; *P* < 0.0001] (Figure 3B). On the other hand, puncturing accompanied by feeding behavior would always lead to the mouthparts rather than the ovipositor being entrapped [*F*_(2, 33)_ = 8.02; *P* < 0.01] (Figure 3C). No significant differences among the three major areas (legs, mouthparts, ovipositor) were noted during capture events involving walking/probing and fighting between leafminer flies [walking/probing: *F*_(2, 39)_ = 2.40, *P* = 0.10; fighting: *F*_(2, 39)_ = 1.93, *P* = 0.16] (Figures 3D,E).

Once the flies became entrapped, they begin to violently struggle rather than passively awaiting death (Video S2). Our results indicated that the escape frequencies of flies captured by landing (50.72 ± 4.26%), walking and probing (66.37 ± 12.42%) were lower than in the other capture behaviors [*F*_(4, 54)_ = 9.36, *P* < 0.0001] (Figure 2C). The escape rate was higher when the ovipositor was captured than when the mouthparts and legs were involved [*F*_(2, 39)_ = 5.43, *P* < 0.01] (Figure 2D).

### Captured body parts

#### Trapped mouthparts

The flies' sponging mouthparts, which appeared to be their most susceptible body part to surface trichomes (Figure 2B), were mostly entrapped when landing and feeding [*F*_(4, 64)_ = 22.79, *P* < 0.0001]. We noted that the labellum, which is traversed by a multitude of pseudotracheae, was commonly trapped by trichomes (Video S3).

#### Trapped legs

**Figure 4:**
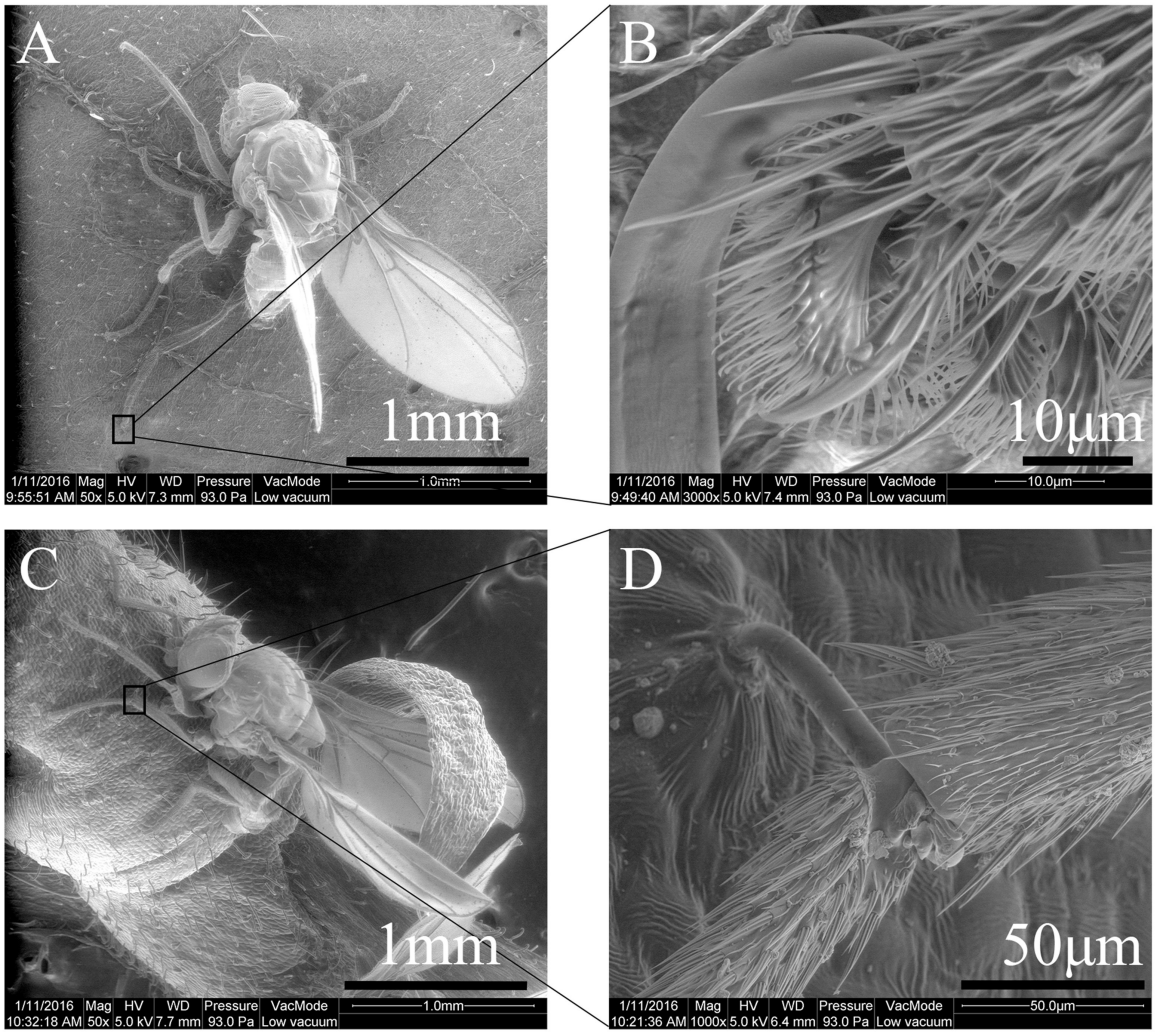
Legs of a *Liriomyza trifolii* adult captured by *Phaseolus vulgaris* surface trichomes: **(A)** *Liriomyza trifolii* adult being trapped on ventral leaf surface, **(B)** trichome impaling pre-tarsus of hind leg. **(C)** *L. trifolii* adult being trapped on stem, **(D)** trichome impaling the intersegmental membranes between femur and tibia of middle leg.

Landing behavior frequently resulted in the trapping of the fore, middle and hind legs of the leafminer flies [*F*_(4, 64)_ = 21.74, *P* < 0.0001] (Videos S1, S2). Trichomes would physically impale the legs between the various leg segments (Figure 4). The susceptible parts of the leg appeared to be the pre-tarsi (Figure 4B) and intersegmental membranes (Figure 4D).

#### Trapped ovipositor

The ovipositors of the females were the second most commonly entrapped body part (Figure 2B), due to their frequent puncturing behavior while attempting to feed or oviposit [*F*_(4, 58)_ = 11.54, *P* < 0.0001]. Unlike the soft mouthparts, the ovipositors of the leafminer females are enclosed in a sclerotized egg-laying tube used to penetrate the leaf surface. Observations from optical microscopy showed that the soft tissues inside the tube (i.e., terminalia), which, when everted during the act of puncturing, were entrapped by the surface trichomes (Video S3).

## Discussion

Careful observation of the behavior of herbivores is the only means of determining the exact capturing sequences of trichome-based plant defenses (Vermeij, 2015). The traditional views on this subject have always assumed that insects were caught simply by crawling or walking across the trichomes (Gilbert, 1971; Levin, 1973; Eisner et al., 1998; Cardoso, 2008). Based on this simplistic assumption, detailed and quantitative studies involving the precise procedure are lacking (Riddick and Simmons, 2014). Our observation showed that the behaviors underlying the trichome-based trapping system were considerably more complex than previously assumed. We were able to define five distinct behavior patterns resulting in the capture of *L. trifolii* adults: landing, puncturing + feeding, puncturing, walking/probing, and fighting (Video S1), with the process of landing contributing to the most entrapments according to this study, although they were also caused, to a lesser extent, by puncture with the ovipositor and feeding behavior and occasionally by walking, or fighting for food or for females.

Landing accounted for most capture events in adult *L. trifolii*. Leafminer flies spend a large percentage of their adult life flying in search of suitable hosts or mates (Parrella, 1987). During the process of landing, different parts of the fly could be entrapped by plant trichomes. Other behaviors such as puncturing + feeding, also resulted in a high level of entrapments. To initiate a puncture of the ovipositor, the females would typically bend the tip of the abdomen down using the ovipositor to make a series of rapid to slow thrusts, finally they would either begin oviposition or return to feeding behavior (Parrella, 1987). The entrapment would frequently occur in the course of slower thrusts (when the egg-laying tissue would be extended), or when the female resumed feeding behavior. In the former situation, the ovipositor would become entrapped; in the latter, the mouthparts would be trapped.

The legs and abdomen are the most widely reported trapped body parts in most trichome-based plant defense systems (Riddick and Simmons, 2014). However, the mouthparts and ovipositor, which have not previously been reported in other insect species, were more frequently entrapped in *L. trifolii* adults. The entrapped leafminer flies, with their body parts hooked on trichomes, were usually prevented from feeding, walking or puncturing. Once firmly entrapped, the flies begin to struggle intensely attempting to free themselves. If they are not successful, they eventually die from starvation and/or dehydration; but even those that are fortunate enough to manage an escape have their fitness substantially reduced due to the injuries suffered and the energy expended during the escape (Johnson, 1953; Pillemer and Tingey, 1976).

The capture efficiency of hooked trichomes was thought to be determined by their density and the insertion angle of the trichomes. Both of these variables are dependant on the plant cultivar (Johnson, 1953; Pillemer and Tingey, 1976). For bean plants, capture mortalities in leafhoppers ranged from 0 to 36.8% depending on the plant cultivar (Pillemer and Tingey, 1976). According to the present study, the leaf stage was the most effective in capturing the leafminer flies, and the ventral side of the leaf was more efficient than the dorsal surface, stem and beanpod. The ventral surfaces (45.4 ± 3.5 [mean ± s.e.m.] trichomes per mm^2^, *n* = 10) of the compound kidney bean leaves have a greater concentration of trichomes than other structures of the plant (dorsal leaf surface: 1.4 ± 0.3 per mm^2^, *n* = 10; stem: 11.5 ± 1.1 per mm^2^, *n* = 10; and beanpod: 10 ± 0.6 per mm^2^, *n* = 10) [*F*_(3, 36)_ = 107.6, *P* < 0.0001], indicating that trichome density does play a significant role in capture effectiveness on this surface. This finding agrees with several previous studies (Gannon and Bach, 1996; Lam and Pedigo, 2001; Björkman and Ahrné, 2005; Economou et al., 2006; Soroka et al., 2011). Since the number of trichomes is fixed early during a plant's development (Pillemer and Tingey, 1976), the densities of hooked trichomes decrease as leaves expand (Stenglein et al., 2004). The highest capture mortality during the leaf stage suggests that trichomes are most effective during that stage of their development. These results indicate that the capture efficiency, in addition to being highly correlated with trichome density as reported in previous studies, was also dependant on trichome development during growth of the plant. In conclusion, the capture efficiency of the trichome-based plant defense system was stage-specific (plant stage) and density-dependent (density of trichomes).

The surface of terrestrial plants plays a crucial role in plant-insect interactions, impacting the behavior of herbivores and their natural enemies in higher trophic levels (Southwood, 1986; Peterson et al., 2016). Results in this study revealed that the surface trichomes found on *P. vulgaris* confer resistance against *L. trifolii* adults by physically trapping their mouthparts, legs and ovipositor, which, in turn, hinder the fly's ability to feed, walk and oviposit. In addition, the entrapments were dependant on trichome development stage and density. These behavioral interactions between the plant surface and herbivores have important implications for pest resistance in crops through traditional or genetic manipulation of the effectiveness and production of additional plant trichomes. From a practical standpoint, a trichome-based physical defense against bed bugs has recently inspired the microfabrication of biomimetic surfaces for eco-friendly control of insect pests (Szyndler et al., 2013). It is hoped that this approach will encourage future biomimetic studies, and further enrich our knowledge of plant-herbivore interactions.

## Author contributions

ZX and ZL: designed the experiments; ZX: performed the experiments; ZL: contributed the materials; YL, XH, SW, and WC: helped analyze the data; ZX: wrote the main manuscript text. All authors read and approved the final manuscript.

### Conflict of interest statement

The authors declare that the research was conducted in the absence of any commercial or financial relationships that could be construed as a potential conflict of interest. The reviewer BB and handling Editor declared their shared affiliation.

## Supplementary material

Video S1 Video recording of different trapping behaviors: landing, puncturing and feeding, puncturing, walking, probing, fighting for females and fighting for food, using a continuously monitoring system.

Video S2 Video recording of different trapped fly body parts, where the flies are still alive and have resumed struggling, using a continuously monitoring system.

Video S3 Video recording of trapped mouthparts and ovipositor in detail, in which the trichomes discontinuously could be seen impaled into each body part, using optical microscopy. Arrows point to the entrap trichome on each body part.
